# Study of laboratory staff’ knowledge of biobanking in Côte d’Ivoire

**DOI:** 10.1186/s12910-020-00533-y

**Published:** 2020-09-11

**Authors:** Ambroise Kouamé Kintossou, Mathias Kouamé N’dri, Marcelle Money, Souleymane Cissé, Simini Doumbia, Man-Koumba Soumahoro, Amadou Founzégué Coulibaly, Joseph Allico Djaman, Mireille Dosso

**Affiliations:** 1 Biobank, Pasteur Institute of Côte d’Ivoire, 01 BP 490 Abidjan, Côte d’Ivoire; 2 Training and Research Unit of Biosciences, Felix Houphouët Boigny University, Abidjan, Côte d’Ivoire; 3 Department of Epidemiology and Clinical Research, Pasteur Institute of Côte d’Ivoire, Abidjan, Côte d’Ivoire; 4 Training and Research Unit of Medical Sciences, Felix Houphouët Boigny University, Abidjan, Côte d’Ivoire

**Keywords:** Biobank, Knowledge, Laboratory staff, Côte d’Ivoire

## Abstract

**Background:**

A biobank is a structure which collects and manages biological samples and their associated data. The collected samples will then be made available for various uses. The sharing of those samples raised ethical questions which have been answered through specific rules. Thus, a Biobank functioning under tight ethical rules would be immensely valuable from a scientific and an economic view point. In 2009, Côte d’Ivoire established a biobank, which has been chosen to house the regional biobank of Economic Community of West African States (ECOWAS) countries in 2018. To ensure optimal and efficient use of this biobank, the scientific community must be aware of its existence and its role. It was therefore necessary to evaluate the knowledge of laboratories staff on the role and activities of a biobank.

**Methods:**

This descriptive study was done by questioning staff from laboratories working on human’s health, animals or plants. The laboratories were located in southern Côte d’Ivoire.

**Results:**

A total of 205 people completed the questionnaire. Of these 205 people, 34.63% were biologists, 7.32% engineers, 48.78% technicians and 9.27% PhD students. The average length of work experience was 10.11 ± 7.83 years. In this study, 43.41% of the participants had never heard of biobanking. Only 48.78% of participants had a good understanding of the role of a biobank. Technicians and PhD students were less educated on the notion of biobank (*p* < 0.000001). Although biologists were more educated on this issue, 21.13% of them had a misconception of biobank. Good knowledge of the role of a biobank was not significantly related to the work experience’s length (*p* > 0.88).

**Conclusion:**

The level of knowledge of laboratory staff about biobanking needs to be improved. Training on the role, activities and interests of the biobank is important.

## Background

Recent advances in the biological and medical fields have increased the value of research on biological samples stored in biobanks. These biological resources are an essential element to the development of research in many developing countries. This research should contribute to improve health care and reduce disease in these countries [1, 2].

A biobank is defined as a structure that receives, processes, stores and makes available biological samples with their associated data for research and clinical care purposes. Samples can be from humans, animals, plants or the environment [3–6].

Among its various roles, the biobank guarantees the quality of the procedures for collecting, transporting, processing, storing and making available biological samples in compliance with the rules of good practice and regulations [6].

Over the past three decades, technological advances have enabled biobanks to grow. As a result, the number of samples stored has increased significantly and biobanks have moved from project-specific (individual) to population-based studies [4, 7].

Today, the biobank is essential for the development of scientific research and is valuable in many ways: (i) it plays a crucial role in the conservation of species [8, 9]; (ii) it contributes to the general health of the population by providing a better understanding of the specific profile of each patient in order to develop better targeted treatments [10]; (iii) it makes it possible to design more effective and less costly drugs [11, 12].

Most biobanks are located in developed countries, but the landscape is changing rapidly as developing countries are increasingly establishing their own biobanks [7, 13–15]. Côte d’Ivoire is among those developing countries. It established a biobank in 2009. Since 2018, that biobank has been housing the regional biobank of the countries of the Economic Community of West African States (ECOWAS). The biobank of Côte d’Ivoire is located within the Pasteur Institute of Côte d’Ivoire (PICI); a Biological and Medical Research Center. A great number of samples from various countries in West Africa, including Benin, Togo, Burkina Faso, Mali and Nigeria are kept at the ECOWAS biobank. These preserved samples are of human, animal, plant, environmental and microbial origin. This biobank is an essential element for the epidemiological surveillance of influenza and measles in Côte d’Ivoire. It was also involved in the project PREDICT 2 of the United States Agency for International Development (USAID) which overall objective was to strengthen the capacity of the surveillance system for human and animal diseases in high-risk regions.

The successful operation of a biobank requires comprehensive interactions between the different actors involved in biobanking, including the public, patients, health care providers, government and donors [16, 17]. Public support, understanding and active participation are essential to the survival of biobanking and research in general. Several studies have described the role of the public [18, 19] and patients [20–22] in biobanking, but very few studies have examined the role of health professionals.

Biological and medical laboratories are the gateways for biological samples which will be kept in biobank then used for research. Therefore, a good knowledge of biobanking by the staff of biological and medical laboratories can ensure an effective collect of samples for biobanking and thus the development of innovation in the field of biomedical research. This could be reached with a regular training on the subject of biobanking for laboratory professionals. However, as a first step, a better understanding of the basic knowledge of laboratory professionals about biobanking is essential. Very few studies have assessed knowledge of biobanking among laboratory professionals in general and no such studies were conducted in Côte d’Ivoire in particular. The main objective of this study was to assess the level of knowledge of laboratory professionals about biobanking.

## Methods

This is a descriptive study that took place from May 28 to August 05, 2019 in biological and medical analysis laboratories in the south of Côte d’Ivoire. Its objective was to assess the level of knowledge of laboratory staff in biobanking. The targeted population was laboratory staff (biologists, engineers, technicians and PhD students (postgraduate students)). Laboratories were selected using a sampling technique based on a pre-determined sampling frame. This database, composed of 450 functional laboratories in southern Côte d’Ivoire, was obtained from the Directorate of Health Facilities of the Ministry of Health and Public Hygiene, the Ministry of Agriculture and Rural Development and the Directorate of Veterinary Services of the Ministry of Animal and Fisheries Resources. From this database, the laboratories have been grouped into three classes:

- Human health laboratories: 360
- Animal health laboratories: 53
- Plant health laboratories: 37

Then a couple of laboratories for each class were chosen. The choice was made by considering the level of the technical platforms and also the private or public character of the laboratories. A total of 66 laboratories were selected: 45 human health laboratories, 15 animal health laboratories and 6 plant health laboratories.

All the staff from the chosen laboratories who volunteer to respond to our question during our visit were included in the study.

### Data collection

The data collection was carried out by a previously trained PhD student. A pre-tested and validated questionnaire was used as a tool to collect data on the professional profile and knowledge of the term “biobank”. The questionnaire was completed in 15 min. The questions in the questionnaire focused on knowledge of the term “biobank” and their role, activities, interests and its existence in Côte d’Ivoire (See questionnaire).

### Statistical analysis

Data entry and analysis were performed using EpiData 3.1 software and STATA 11 software respectively.

Statistical tests (Pearson’s chi-2 or Fisher) with a 95% confidence level were used to test the existence of a statistical link between “knowledge of the role and activities of a biobank” and professional profile variables such as “function”, “type of laboratories”, “laboratory characteristics” and “types of activities” in the laboratories where the participants came from. Then, the mean comparison test with a 95% confidence level was used for the existence of a statistical link between “knowledge of the role and activities of a biobank” and “the length of work experience”.

### Ethical conditions

Arrangements had been made to ensure that this study is conducted under ethical conditions. Indeed, the research ethics committee of the PICI has given its approval for the realization of this study. Also, the investigation only began after obtaining the authorization of the heads of the selected laboratories. In addition, the information was collected with the consent of the participants and in absolute confidentiality, and made anonymous and encrypted.

## Results

A total of 205 people from 66 laboratories participated in the study. These were 45 human health laboratories, 15 animal health laboratories and 6 plant health laboratories (See Table 1). Of these, 33 were research laboratories and 57 were public laboratories. The respondents were 149 (72.68%) male and 56 (27.32%) female (See Table 1). The average length of work experience was 10.11 ± 7.83 years with extremes ranging from 1 to 36 years. Of the 205 respondents, 50% had more than 8 years of work experience. The workforce was mainly composed of technicians (48.78%) (See Table 1). Staff from public laboratories represented 82.93% of the participants. Those from research laboratories and human health laboratories represented 49.27 and 70.73% of participants respectively. The PICI laboratory staff included in this study represented 30.24% of respondents.

**Table 1:** Professional profile of respondents

Characteristics	Staff size	Percentage (%)
**Length of work experience**		
1–5 years	78	38.05
6–10 years	50	24.39
> 10 years	77	37.56
**Gender**		
Male	149	72.68
Female	56	27.32
**Respondents functions**		
Biologists	71	34.63
Biology Engineers	15	7.32
Biology Technicians	100	48.78
PhD Students	19	9.27
**Respondents laboratories types**		
Human health	145	70.73
Animal health	46	22.44
Plant health	14	6.83
**Respondents laboratory statuses**		
Public	170	82.93
Private	35	17.07
**Respondents from research center laboratories and others**		
Research centers	101	49.27
Others centers	104	50.73

In this study, 56.23% of participants reported that they had already heard of biobank (See Table 2). Among these, the staff from the PICI had all heard of biobank, while 62.23% of the staff from the other laboratories surveyed had never heard of biobank. The majority of respondents heard about biobank for the first time in their work (32.68%) and training courses (21.46%) (See Table 2). To the question: What are the role and activities of a biobank (See questionnaire); 48.78% gave an exact answer. That is to say, collecting, preserving and making available samples with their associated data. Of these, 2% were unaware of the importance of traceability of biological samples in the biobanking process. As for the biobank of Côte d’Ivoire, 44.88% knew about its existence and location. As for the regional biobank of ECOWAS countries, 63.41% were unaware of its existence (See Table 2). Six of the respondents stated that they use biobanks other than the one in Côte d’Ivoire for the conservation of their biological samples. These were the biobanks of Europe and North Africa. The bi-varied analysis showed that technicians and PhD students were less educated on the notion of biobank (*p* < 0.000001). 21.13% of biologists, although they were more educated on this issue, have a misconception of biobank. Good knowledge of the role of a biobank was not significantly related to the length of work experience (*p* > 0.88). But it was significantly related to the type of laboratory, the character of the laboratory and the type of activity of the laboratory. Indeed, laboratory staff who had a better understanding of the role of a biobank were from animal health laboratories (*p* < 0.002), research laboratories (*p* < 0.000001) and public laboratories (*p* < 0.006) (See Table 3). Staff from PICI laboratories had a better knowledge of the role of a biobank than staff from other laboratories (*p* < 0.000001). However, 8.06% of them had an erroneous notion (See Table 3).

**Table 2:** General knowledge about biobank

Variables	Staff size	Percentage (%)
**Have you ever heard of biobank?**		
Yes	116	56.23
No	89	47.77
**Where have you heard about biobank before?**		
Mass media	5	2.44
At work	67	32.68
During a training session	44	21.46
Never heard of it	89	43.41
**Role and activities of a biobank**		
Correct answer	100	48.78
Wrong answer	16	7.80
Don’t know	89	43.42
**In your opinion, does sample traceability play a role in the biobanking process?**		
Yes	96	46.83
No	16	7.8
Don’t know	93	45.37
**Did you know that Côte d’Ivoire has a biobank and do you know where it is located?**		
Yes	92	44.88
No	113	55.12
**Did you know that Côte d’Ivoire also has an ECOWAS regional biobank and do you know where it is located?**		
Yes	75	36.59
No	130	63.41

**Table 3:** Association between knowledge of the definition, role and activities of a biobank and professional profile

Professional profile	Definition, role and activities of a biobank				***P*** value
	Correct answer		Wrong answer		
	n	%	n	%	
**Functions**					
Biologists	56	78.87	15	21.13	< 0.000001
Biology Engineers	6	40	9	60	
Biology Technicians	31	31	69	69	
PhD Students	7	36.84	12	63.16	
**Respondents from different types of laboratories**					
Human health	55	37.93	90	62.07	< 0.002
Animal health	37	80.43	9	19.57	
Plant health	8	57.14	6	42.86	
**Respondents from different laboratory statuses**					
Public	90	52.94	80	47.06	< 0.006
Private	10	28.57	25	71.43	
**Respondents from research center laboratories**					
Yes	83	82.18	18	17.82	< 0.000001
No	17	16.35	87	83.65	
**Respondents from PICI laboratories and others**					
PICI laboratories	57	91.94	5	8.06	< 0.000001
Others laboratories	43	30.07	100	69.93	

## Discussion

The African continent has the highest number of epidemics (more than 100 per year). Preparedness, surveillance and response to health crises are essential for the successful implementation of the International Health Regulations. With this in mind, the World Health Organization (WHO) has asked PICI to confine poliovirus, yellow fever, influenza and HIV specimens. Thus, in 2009, Côte d’Ivoire and the PICI decided to create a biobank with international standards. This project had three components: rehabilitation of existing structures, construction of a cryobiology room, training of the staff in Europe and the purchase of state-of-the-art equipment to offer a various range of conservation techniques. This state-of-the-art equipment for the conservation of high-quality biological samples resulted in the PICI biobank to be chosen as the biobank of the ECOWAS countries. This biobank participates in national and international research programs, notably in the epidemiological surveillance of influenza and measles in Côte d’Ivoire. As well as in the USAID PREDICT 2 project which general objective was to strengthen the capacities of the human and animal disease surveillance system in high-risk regions.

In this context, the emergence of a biobank as a structure of sample collections combined with relevant patient information requires the active participation of the public, patients, health care providers, government and donors. Several studies have described the role of the public [18, 19] and patients [20, 21] in biobanking, but very few studies have examined the role of health professionals.

Biomedical laboratories are the gateways for biological samples. Therefore, education and awareness of laboratory professionals is paramount to ensure the proper management of high-quality biological samples and the collection of real-time patient information. An initial assessment of knowledge about biobanking in Côte d’Ivoire is a prerequisite for the development of appropriate awareness programs for the public, patients or health care providers.

In our study, we sought to determine the level of knowledge of laboratory professionals about biobanking. We present the results of this survey conducted in biomedical analysis laboratories in the south of Côte d’Ivoire. The study showed that 56.59% of the participants had heard of the term “biobanking” before. It should be noted that laboratory personnel from PICI, the structure housing the biobank in that country, were included in the study and represented 30.24% of the participants. They had all stated that they had already heard of biobanking.

Sixty-two point 23 % of the non-PICI laboratory staff had never heard of biobanking. This is lower than in the study by Merdad et al. where 73% of health science students in Saudi Arabia reported never having heard of biobanking [23]. This limited knowledge is consistent with the results of previous surveys conducted in other parts of the world [24–28].

The level of knowledge about the role and activities of biobanks among the surveyed laboratory staff is low, even including PICI laboratory staff. Indeed, 8.06% of PICI laboratory staff compared to 69.93% of other laboratory staff is unaware of the exact role and activities of a biobank. This is in contrast to a study among Italian students where 83.7% were aware of the role and activities of biobanks [29]. The results also showed that biologists had significantly higher knowledge of the role and activities of a biobank than engineers, technicians and PhD students. This may be explained by the fact that most of the biologists surveyed are researchers and are more involved in scientific training. Among the people surveyed, 2.93% stated that they use the service of biobanks located in Europe and North Africa for the conservation of their biological samples. This translates into the fact that they are unaware of the existence of a biobank in Côte d’Ivoire.

This limited knowledge seems to be linked to the novelty of the concept in Côte d’Ivoire, but also to the fact that the term itself can be confusing for many people. Indeed, during the survey, some people defined biobanks as savings structures reserved for biologists.

## Conclusion

The current study was carried out to address the lack of awareness about biobanking in Africa, specifically in Côte d’Ivoire, through a customized questionnaire for laboratory professionals. The results showed a notable lack of awareness of the term “Biobank” and its role, activities, interests and existence in Côte d’Ivoire. There is therefore a need to plan additional awareness training programs tailored to laboratory professionals to familiarize them with biobanking. The awareness of the importance of active participation in biobanking should also be extended to all biobank staff, health care providers, including laboratory professionals, and the general public in order to improve sampling collection and thus enhance the value of biomedical research in Côte d’Ivoire and even in Africa.

## Supplementary information

**Additional file 1.** Questionnaire.

## Data Availability

The datasets used and/or analyzed during the current study are available from the corresponding author on reasonable request.

## Abbreviations

PICI: Pasteur Institute of Côte d’Ivoire
ECOWAS: Economic Community of West African States
WHO: World Health Organization
USAID: United States Agency for International Development
