# Supplementary material for: Study of laboratory staff’ knowledge of biobanking in Côte d’Ivoire
Source: BMC Med Ethics. 2020 Sep 11;21:88. doi: 10.1186/s12910-020-00533-y (PMC7488401; doi:10.1186/s12910-020-00533-y)
Supplement: Supplementary file 1 — Additional file 1. Questionnaire. [file 12910_2020_533_MOESM1_ESM.docx]

**QUESTIONNAIRE**

/___/___/___/----/___/___/

District Code Order Number

Section I: SOCIO-DEMOGRAPHICAL CHARACTERISTICS

| **N°** | **QUESTIONS** | **RESPONSES** | **CODE** |
| --- | --- | --- | --- |
| QU101 | Gender of respondent | - 1. Male - 2. Female |  |
| QU102 | What is your function in your department? | - 1. Physician - 2. Pharmacist - 3. Scientist - 4. PhD student - 5. Laboratory engineer - 6. Laboratory technician |  |

Section II : GENERAL KNOWLEDGE ON BIOBANKING

| **N°** | **QUESTIONS** | **RESPONSES** | **CODE** |
| --- | --- | --- | --- |
| QU201 | Have you ever heard of biobanking? | - 1. Yes - 2. No |  |
| QU202 | Where did you hear about the  first time in biobanking? | - 1. The media (radio, TV) - 2. At a relative's home - 3. With a health worker - 4. During a seminar - 5. During a training course - 6. On the net - 7. Other (specify) - 8. Never |  |
| QU203 | What do you think a biobank is? | - 1. space for collections of human samples and associated data. - 2. Space for collections of biological samples and associated data. - 3. Human specimen collection space - 4. Hospital - 5. Research Center - 6. Space for coin collections and management - 7. Sector of economic activity - 8. Museum - 9. Don't know |  |
| QU204 | What are the activities carried out by a biobank? | - 1. Credit distribution and deposit collection - 2. Reception, processing, storage and provision of biological samples - 3. Securing sample data - 4. Preservation of biological samples - 5. Don't know |  |
| QU205 | In your opinion, does traceability play a role in the biobanking process? | - 1. Yes - 2. No - 3. Don’t know |  |
| QU206 | Did you know that Côte d'Ivoire has a biobank? | - 1. Yes - 2. No - 3. Don’t know |  |
| QU207 | If so, do you know its location? | - 1. Yes - 2. No - 3. Don’t know |  |
| QU208 | Did you know that Côte d'Ivoire also has an ECOWAS biobank? | - 1. Yes - 2. No - 3. Don’t know |  |
| QU209 | If so, do you know its location? | - 1. Yes - 2. No - 3. Don’t know |  |
